# ALARA in Urology: Steps to Minimise Radiation Exposure During All Parts of the Endourological Journey

**DOI:** 10.1007/s11934-022-01102-z

**Published:** 2022-08-13

**Authors:** Radhika Bhanot, Zeeshan B. M. Hameed, Milap Shah, Patrick Juliebø-Jones, Andreas Skolarikos, Bhaskar Somani

**Affiliations:** 1 Department of Urology, University Hospital Southampton, Southampton, UK; 2 Department of Urology, Kasturba Medical College Manipal, Manipal Academy of Higher Education, Manipal, Karnataka India; 3 Dept of Urology, Haukeland University Hospital, Bergen, Norway; 4 Department of Urology, National and Kapodistrian University of Athens, Athens, Greece; 5 Department of Urology, University Hospital Southampton NHS Trust, Southampton, UK

**Keywords:** ALARA, Radiation, Ureteroscopy, Kidney calculi, Percutaneous nephrolithotomy, Radiation, Safety, Endourology

## Abstract

**Purpose of the Review:**

The global burden of kidney stone disease (KSD) and its management relies on ionising radiation. This includes the diagnosis, treatment and follow-up of KSD patients. The concept ‘As Low As Reasonably Achievable’ (ALARA) developed in response to the radiation risks and the key principles include optimisation, justification and limitation of radiation. This article provides an overview of the topic including background to the risks and steps that can be taken during all stages of endourological management.

**Recent Findings:**

Our review suggests that ionising radiation is an invaluable tool in delineating the anatomy, localising disease, guiding manoeuvres and monitoring treatment in patients with KSD. It therefore plays an integral role in many stages of patient care; preoperatively, intraoperatively and postoperatively.

The reduction of radiation pre- and post-surgical intervention relies on the use of low-radiation CT scan and ultrasound scan. It can also be achieved through various intraoperative techniques or fluoroless techniques in selected patients/procedures, customised to the patients and procedural complexity.

**Summary:**

There are many parts of the patient journey where exposure to radiation can take place. Urologists must be diligent to minimise and mitigate this wherever possible as they too face exposure risks. Implementation of strategies such as teaching programmes, fluoroscopy checklists and judicious use of CT imaging among other things is a step towards improving practice in this area.

## Introduction

Ionising radiation continues to play a central role in the diagnosis, treatment and follow-up of many urological conditions [1•]. Non-contrast computed tomography (NCCT) scan is the gold-standard imaging modality for diagnosing kidney stone disease (KSD) and reports show that the number of CT scans performed for this condition increased tenfold between 1996 and 2007 [2, 3]. Fluoroscopy is another important tool in the endourological armamentarium, and this leads to radiation exposure for both patient and operating team including anaesthesiologists, nurses and other ancillary staff. Given the increasing global burden of KSD and the subsequent rise in endourological interventions being performed, the level of radiation exposure is expected to mirror this accordingly [4–6]. This is concerning given the known risks associated with such exposure to radiation, including malignancy. Up to 2% of new cancers diagnosed in the USA are estimated to be attributed to CT imaging [7]. The concept ‘As Low As Reasonably Achievable’ (ALARA) developed in response to these risks and the key principles include optimisation, justification and limitation of radiation [8•]. This article provides an overview of the topic including background to the risks and steps that can be taken during all stages of endourological management.

### Risks of Radiation

In order to appreciate the risks of radiation, a basic understanding of the mechanism and measurements of radiation exposure is useful. High-energy photons (otherwise known as X-rays) can break molecular bonds and ionise atoms, leading to the production of free radicals, which can induce DNA damage [9]. The total radiation emitted encompasses both direct exposure from the X-ray beam and inadvertent scattered X-rays. There are three methods of expressing radiation doses: the absorbed, equivalent and effective doses.

Radiation exposure can be measured by quantifying the energy absorbed per unit of body mass, expressed in milligreys (mGy) (Table 1). If exposed to the same dose, different organs will absorb variable amounts of radiation and so the millisievert (mSv) unit is used to estimate the equivalent biological dose of this radiation. The effective radiation dose takes both independent organ sensitivity and overall risk to the recipient into consideration by adding together the individual organ equivalent doses. Radiation-induced injury is classified as deterministic or stochastic. Deterministic effects are dictated by the lifetime equivalent, i.e. the cumulative exposure received over time. This relies on a certain threshold dose of radiation to have been reached. These dose-dependent effects increase in severity as the dose increases beyond the threshold. These are exerted through direct cell killing and include dermal injury, cataract formation, thyroiditis, bone marrow suppression and hair loss.

Stochastic effects, on the other hand, do not require a threshold dose to be met. They can lead to respiratory, cardiovascular and gastrointestinal diseases, which can often present several years following radiation exposure. Furthermore, the severity of effects remains independent of the dose. Radiation-induced malignancy is one of the more concerning stochastic effects and appears to follow a linear, no-threshold hypothesis (LNTH) [10]. This model explains that any level of radiation exposure, including low dose, carries a potential risk and may still contribute to cancer development. Preston et al. found an association with solid and haematological malignancies in cases with doses as low as 5 mSv [9].

### Guidelines

The International Commission on Radiological Protection (ICRP) has issued guidelines on safe occupational radiation exposure for healthcare workers, but not for patients [11]. A safe limit of 20 mSv per annum is given for a maximum duration of 5 years or 50mSV of radiation in a single year. This is equivalent to 2 to 3 CTs of the abdomen and pelvis or 7 to 9 years of background radiation. Evidence reveals exposure beyond this limit is associated with a 1 in 1000 lifetime risk of fatal cancer [12].

### Strategies to Reduce and Mitigate Radiation Exposure Among Patients

Ionising radiation is an invaluable tool in delineating the anatomy, localising disease, guiding manoeuvres and monitoring treatment in patients with KSD. It therefore plays an integral role in many stages of patient care: preoperatively, intraoperatively and postoperatively.

#### Preoperatively

Arguably, the best strategy to minimise exposure preoperatively is through diligent patient selection and stringent review of every clinical indication for using radiation. Patient education is important and involves conveying the risks associated with imaging to the patient and balancing this with its value addition in the clinical setting. In a prospective study by Busey et al., only 3% of patients reported having thought about the radiation they were going to be exposed to as part of their diagnostic imaging [12].

While an increasing number of institutions worldwide have adopted strategies to identify and document the pregnancy status of patients prior to receiving radiation, most do not require evidence confirming that the risks of radiation have, in fact, been communicated to the patient [13]. The role of a health professional acting as a radiation steward in this setting could be helpful for counselling purposes. The role of a ‘radiation passport’ is another novel solution, whereby patients’ exposure dose levels are recorded on a document, also available as a smartphone application, which the patient takes to each imaging appointment for clinicians to monitor the cumulative dose received [14].

While diagnostic imaging may be unavoidable in some cases, alternatives such as ultrasound should be considered. Although these are less sensitive for KSD, it does reduce exposure. Current guidelines recommend non-contrast CT KUB as the gold-standard imaging in diagnosing renal/ureteric calculi [2] (Table 1). The radiation risk is reduced with ‘low-dose’ CT, which has 93.1% sensitivity and 96.6% specificity [15•].

In pregnancy, radiation exposure may lead to stochastic and teratogenic sequelae. Guidelines advise US and MRI (magnetic resonance imaging) as first- and second-line investigations respectively [2]. While low-dose CT is associated with a higher positive predictive value (95.8%) compared to MRI (80%) and ultrasound (77%) in diagnosing urolithiasis in pregnancy, its use is generally reserved as the last option [16]. In the paediatric setting, EAU (European Association of Urology) guidelines recommend US as the first line of investigation followed by low-dose NCCT [2].

#### Intraoperatively

Intraoperatively, there are three main technical factors, which affect radiation levels: duration of exposure, distance from the X-ray beam and physical shielding (Table 2).

As a general rule, duration of exposure should be minimised, to only what is absolutely necessary. This can be executed through employment of trained ‘ALARA-conscious’ technicians or radiographers, pre-procedure planning, accurate patient positioning and avoidance of magnification unless clinically required. It is worth noting that standard fluoroscopy takes around 35 images per second [8•]. In contrast, pulsed fluoroscopy decreases the exposure, taking around 5 images per second, without compromising image quality [17].

The shorter the distance between the patient and the radiation source, the higher both direct and scattered radiation exposure. Giordano et al. confirmed these findings by demonstrating a more than double dose increase when a cadaver was placed closest to the radiation source rather than closest to the image intensifier (or 10 in. away from the radiation source) [18]. Image intensifiers, which should be positioned as near as possible to the patient, act to reduce the exposure and produce high-quality images. Alarms can be used on the C-arm to alert operator to fluoroscopy time.

In preparation for any operative case using radiation, a dedicated member of the team should monitor the amount of radiation the patient is receiving and feedback to the operator once the limit has been reached. Physical shielding involves standing behind a physical barrier and/or wearing shields/aprons and protective eyewear. Theatre staff should assess which of the patient’s body parts should be shielded and these shields must be kept away from the path of the radiation so as to avoid disrupting the X-ray image.

Increasingly, outcomes from low-dose fluoroscopy as well as fluoroless endourological procedures are being reported [19]. The latter implement measures such as tactile guidance and even US to avoid fluoroscopy [8•].

### Strategies to Reduce Radiation Exposure Among Urologists and the Healthcare Team

One of the main ways staff can minimise radiation exposure is by only using radiation where the benefits outweigh the risks. Research has shown that during diagnostic procedures, 70–97% of urologists underestimate the radiation exposure received by patients [20]. Staff training in ALARA principles and education in radiation safety should be standardised and a compulsory part of induction programmes to promote judicious use of radiation. Friedman et al. reported that over 40% of urology residents failed to receive adequate radiation safety training [21].

The correlation between fluoroscopy time and radiation exposure has been well established and Frederick-Dyer et al. achieved a 25% reduction in fluoroscopy time following staff training [22].

The key difference in exposure between staff and patients is the source of radiation. While patients typically face the majority of exposure directly from the source beam, healthcare staff are often more susceptible to scattered radiation which includes X-rays emitted from the patient via photoelectric effect and Compton scattering [23]. This can be mitigated by staff increasing their distance from the X-ray source. The onus lies with the operator to warn theatre staff when the radiation is about to be activated, and to ensure all personnel are wearing personal protective equipment (PPE).

In the context of PCNL (percutaneous nephrolithotomy) procedures, multiple studies have found an association between increased radiation exposure and increased stone burden, operative time, BMI and multiple access tracts [24, 25] (Table 2).

A reduction in radiation exposure can be achieved through various intraoperative techniques. These include the use of pulsed fluoroscopy with as low as possible pulse rate (frames per second), use of air instead of contrast during retrograde studies, having a last image hold, employing an under-couch radiation source instead of over-couch, using ultrasound guidance for access procedures and using image collimation to restrict the irradiated area [8•, 25]. These techniques can also reduce the scattered radiation, thus minimising exposure to the theatre staff.

Implementation of a pre-fluoroscopy checklist has been shown to reduce total fluoroscopy time by 67% [26]. Alerting staff to wear PPE, allowing the operator time to clarify the collimation, adjusting the beam size, confirming the patient position and checking the pregnancy status are all steps to minimise unwanted radiation exposure.

The different types of shields include ceiling-suspended, portable rolling and lead-based garments, which may be either front aprons, vest and skirt or wraparounds which confer greater coverage for protection. Evidence suggests that portable rolling mobile shields can reduce the effective radiation dose by over 90% [27].

Lead aprons exist in various thicknesses and allow a radiation transmission of up to 5% [1•]. The size of the apron should be based on the manufacturer’s sizing chart. These require cleaning in between uses, regular (6-monthly) checks and when not being used should be hung rather than folded to avoid cracking and loss of integrity. Thyroid shields may limit the radiation exposure from 46 to 0.02 mSv [11]. Studies have investigated causes for poor compliance and have found this to be due to orthopaedic issues including pain resulting from the weight of aprons as well as lack of standardised education programs [21].

Ideally, dosimeters should be worn both inside and outside of the lead apron to allow comparison of the cumulative occupational radiation exposure. Dosimeters provide objective feedback to staff, which may help raise safety awareness and encourage ALARA-conscious behaviours to reduce risk. However, in a survey of urology residents, Harris et al. reported that only 30% use dosimeters [28].

Extra guidance is required for staff who are pregnant. However, a recent study revealed that in Europe, there are numerous countries where none exists [29]. Steps to reduce radiation must be customised to the patients and procedural complexity [30•, 31–36]. Perhaps the use of artificial intelligence can also help achieve this goal [37].

## Conclusion

There are many parts of the patient journey where exposure to radiation can take place. Urologists must be diligent to minimise and mitigate this wherever possible as they too face exposure risks. Implementation of strategies such as teaching programmes, fluoroscopy checklists and judicious use of CT imaging among other things is a step towards improving practice in this area.
