# Correction: ALARA in Urology: Steps to Minimise Radiation Exposure During All Parts of the Endourological Journey

**DOI:** 10.1007/s11934-022-01111-y

**Published:** 2022-10-01

**Authors:** Radhika Bhanot, Zeeshan B. M. Hameed, Milap Shah, Patrick Juliebø-Jones, Andreas Skolarikos, Bhaskar Somani

**Affiliations:** 1 Department of Urology, University Hospital Southampton, Southampton, UK; 2 Department of Urology, Kasturba Medical College Manipal, Manipal Academy of Higher Education, Manipal, Karnataka India; 3 Dept of Urology, Haukeland University Hospital, Bergen, Norway; 4 Department of Urology, National and Kapodistrian University of Athens, Athens, Greece; 5 Department of Urology, University Hospital Southampton NHS Trust, Southampton, UK

## Correction to: Current Urology Reports https://doi.org/10.1007/s11934-022-01102-z

In the original version of this article, the given and family names of all authors were incorrectly structured.

The correct names should be:

Radhika Bhanot, Zeeshan B. M. Hameed, Milap Shah, Patrick Juliebø-Jones, Andreas Skolarikos, Bhaskar Somani

The original article has been corrected.

